# Study on correlation between property of coronary artery lesion and degree of coronary artery stenosis of elderly patients with coronary heart disease

**DOI:** 10.12669/pjms.35.1.225

**Published:** 2019

**Authors:** Chao Wang, Xiang Tian, Wei Xia, Qianmei Liu

**Affiliations:** 1 Chao Wang, Department of Cardiology, Baoding First Hospital Baoding 071000, P. R. China; 2 Qianmei Liu, Department of Cardiology, Baoding First Hospital Baoding 071000, P. R. China; 3 Wei Xia, Department of Cardiology, Baoding First Hospital Baoding 071000, P. R. China; 4 Xiang Tian, Department of Cardiology, Baoding First Hospital Baoding 071000, P. R. China

**Keywords:** The old, CHD, Coronary artery lesion, Left ventricular remodeling, Correlation

## Abstract

**Objective:**

To discuss the influence of coronary artery lesion of elderly patients with coronary heart disease (CHD) on left ventricular remodeling.

**Methods:**

Retrospective selection method was used to choose 80 elderly CHD patients who received coronary angiogram examination in Baoding First Central Hospital from January 2014 to February 2018 as the objects of study. According to coronary artery lesion, the patients were classified into single vessel lesion group (single vessel group) and multi-vessel lesion group (multi-vessel group, the number of lesion vessels≧2). Single vessel group included 60 patients, and multi-vessel group includes 20 patients. Intravascular unltrasound was applied to record coronary plaque property of all patients and transthoracic echocardiography was used to record left ventricular remodeling. Later correlation analysis was carried out.

**Results:**

The proportion of calcified plaque and mixed plaque was higher than that of single vessel group, and the differences had statistical significance (P<0.05). Left ventricular end diastolic volume (LVEDV) and left ventricular end-systolic volume (LVESV) of multi-vessel group were higher than that of single vessel group, while left ventricular ejection fraction (LVEF) was lower than that of single vessel group. The differences had statistical significance (P<0.05). Linear correlation analysis showed coronary artery lesion was positively correlated with LVEF and calcified plaque (r=0.287, 0.371, P<0.05). Multiple linear regression analysis showed LVEF, calcified plaque and LDL-C were independent risk factors of multi-vessel coronary artery lesion of old CHD patients (P<0.05).

**Conclusion:**

The number of coronary artery lesions is significantly correlated with left ventricular remodeling, and can increase the proportion of calcified plaque and mixed plaque, thus leading to left ventricular remodeling abnormity.

## INTRODUCTION

With rapid economic development, improvement of living standards in China, accelerated population aging progress and change of life style, both morbidity and death rate of cardiovascular disease rises year by year. Cardiovascular disease has become the second largest disease, only second to malignant tumor. CHD is the major fatal disease.^1^,^2^ Epidemiology shows the morbidity of CHD in China is close to 2%, and the morbidity among the elderly exceeds 5%.^3^,^4^

Modern researches display that CHD patients mainly suffer by the formation of intra-coronary plaque resulting from atherosclerosis in coronary arteries. The development of plaque will result in luminal stenosis and even obstruction will take place. Finally, myocardial ischemia and anoxia will lead to damage of cardiac muscles.^5^-^7^ Myocardial cell damage can reduce cardiac pump function. The heart changes to spherical structure from normal ellipsoid structure, and thus cardiac dilatation occurs, thus leading to ischemic cardiomyopathy.^8^,^9^

Studies have showed that, the formation of vulnerable plaque and occurrence of coronary event are closely related to left ventricular remodeling. Intravascular unltrasound can recognize the property of coronary artery lesion and vascular wall structure of old CHD patients, and judge remolding property of plaque and plaque property so as to predict left ventricular remodeling.^10^,^11^ Early identification of the nature of coronary atherosclerotic plaque and its relationship with left heart remodeling is essential for early intervention in the treatment of coronary heart disease. In this study, the influence of coronary artery lesion of old CHD patients on left ventricular remodeling is discussed to clarify the mechanism of left ventricular remodeling.

## METHODS

This retrospective selection method was used to choose 80 elderly CHD patients who had coronary angiography examination in Baoding First Central Hospital from January 2014 to February 2018 as the objects of study. The study was approved by the Institutional Ethics Committee of our hospitals, and written informed consent was obtained from all participants

### Inclusion criteria

Age≧60; CHD diagnosed by coronary arteriography; complete clinical and diagnosis data; all the patients signed informed consent form; the patients did not take statin or receive lipid regulation and lipid-lowering drug treatment four weeks before hospitalization.

### Exclusion criteria

Patients with infectious diseases, injury and malignant tumor; pregnant women and lactating women; patients with diffuse stenosis or branch and opening stenosis. The comparison differences in gender, age, disease type, course of disease and LVEF of both groups had no statistical significance (P>0.05), as shown in Table-I.

**Table-I:** General data comparison.

Group	No. (n)	Disease type (acute myocardial infarction / unstable angina pectoris / stable angina pectoris)	Course of disease (month)	BMI (kg/m2)	Gender (male/female)	Age (year)	LVEF (%)
Multi-vessel group	20	10/5/5	5.29±1.94	22.74±3.13	11/9	47.20±4.19	54.29±6.15
Single vessel group	60	32/16/12	5.30±2.09	22.18±2.78	33/27	47.13±3.89	54.11±5.82
t or X^2^		0.241	0.045	0.344	0.000	0.144	0.201
P		0.886	0.893	0.571	1.000	0.755	0.714

Mainstream intravascular unltrasound examination equipment manufactured by American VOLCANO was used, with the ultrasonic frequency of 9-14MHz. The patients adopted supine position. The ultrasonic probe was put at the far end of coronary artery lesion for continuous vertical section and cross section examination, and automatically retracted to the near end of lesion at the 0.5mm/s constant speed to record imaging characteristics.

Plaque property: (1) Mixed plaque: if the same section shows multiple kinds of echo, the plaque is mixed plaque; (2) Calcified plaque: echo intensity of the plaque exceeds adventitia echo of coronary artery wall, accompanied with acoustic shadow at the rear of the plaque; (3) Fiber plaque: echo intensity of the plaque is similar to adventitia echo of coronary artery wall; (4) Lipid plaque: plaque echo is lower than adventitia echo of coronary artery wall.^7^

### Parameters of left ventricular remodeling

Acuson S2000 diasonograph of Siemens was chosen, with the ultrasonic frequency of 9-14MHz. SIMPSON method was used to gain LVEDV, LVESV, LVEF and other parameters according to the principle of counting-volumetric method and the count of end-systole and end diastole of cardiac cycle.

Three to five milliliter fasting venous blood before treatment was drawn from the patients and determined by Baoding First Central Hospital. Roche full-automatic biochemical analyzer was used for the test. The test indexes included blood glucose and blood fat.

### Statistical Method

SPSS19.00 software was used to analyze measurement data and enumeration data. Measurement data and enumeration data were expressed with mean ±standard deviation, and percentage. T test and chi-square X2 analysis were applied for contrast. Linear correlation analysis and multiple linear regression analysis were used for correlation analysis. Test level: ɑ=0.05.

## RESULTS

The proportion of fiber plaque and lipid plaque in multi-vessel group was higher than that in single vessel group. The proportion of mixed plaque and calcified plaque in multi-vessel group was lower than that in single vessel group. The differences had statistical significance (P<0.05), as shown in Table-II.

**Table-II:** Comparison of plaque property (n).

Group	No.(n)	Fiber plaque	Lipid plaque	Calcified plaque	Mixed plaque
Multi-vessel group	20	4(20.0%)	4(20.0%)	8(40.0%)	4(20.0%)
Single vessel group	60	7(11.7%)	6(10.0%)	28(46.7%)	19(31.7%)
X^2^		6.955			
P		0.003			

LVEDV and LVESV values in multi-vessel group were higher than that in single vessel group. LVEF value was lower than that in single vessel group. The comparison differences had statistical significance (P<0.05), as shown in Table-III.

**Table-III:** Comparison of left ventricular remodeling indexes (mean±standard deviation).

Group	No.(n)	LVEDV(ml)	LVESV(ml)	LVEF (%)
Multi-vessel group	20	140.69±11.48	85.02±15.20	51.09±8.22
Single vessel group	60	126.30±30.20	62.49±10.88	56.20±7.28
t		13.092	14.592	5.799
P		0.000	0.000	0.011

### Correlation Analysis

Among the patients in both groups, linear correlation analysis showed coronary artery lesion was significantly related to LVEF and calcified plaque (r=-0.287, 0.371, P<0.05). Multiple coronary artery lesion was used as the dependent variable, and clinical investigation data, ultrasonic test index and hematology test data were used as the independent variables. Multiple linear regression analysis displayed that, LVEF, calcified plaque and LDL-C were independent risk factors of multi-vessel coronary artery lesion of old CHD patients(P<0.05), as shown in Table-IV.

**Table-IV:** Multi-factor Logistic regression analysis of multiple coronary artery lesion of old CHD patients (n=178).

Variable	β	SE	Wald	P	OR	95%CI
LDL-C	0.012	0.005	6.109	0.013	2.781	1.194-6.123
Calcified plaque	1.882	0.598	8.813	0.003	3.298	1.284-7.343
LVEF	0.029	0.013	5.098	0.024	3.098	1.333-8.092

## DISCUSSION

CHD refers to a heart disease caused by myocardial anoxia and ischemia resulting from vascular cavity obstruction due to atherosclerosis of coronary artery. CHD is common among the elderly. The number of coronary artery lesions represents the severity of CHD.^12^,^13^

This study shows that the proportion of mixed plaque and calcified plaque in multi-vessel group was higher than that in single vessel group, and the differences had statistical significance. The main compositions of fiber plaque and lipid plaque include lipid, necrotic tissue, fresh thrombus or loose cellular fibrous tissue. The main compositions of mixed plaque and calcified plaque include dense fibrous tissue and calcification composition.^14^ The stiffness of lipid is smaller than that of calcified tissue. Thus, when lumen area decreases, the tissue rich in lipid can dilate more easily under the action of shear stress. When the lesion is in the proliferating phase, more lipid compositions and inflammatory cells infiltrate. Thus, the plaque is unstable, and may fracture easily thus leading to CHD and multiple coronary artery lesion.^15^

Ventricular remodeling refers to myocardial structure, function and configuration changes caused by a series of complex molecule and cell mechanisms. The change of ventricular remodeling in turn deteriorates systolic and diastolic functions and even conduction function of CHD patients. Thus, the degree of left ventricular remodeling is closely related to cardiac function and prognosis.^16^,^17^ This study shows that LVEDV and LVESV values in multi-vessel group were higher than that in single vessel group, while the LVEF value was lower than that in single vessel group. The contrast differences had statistical significance (P<0.05). From the perspective of mechanism, myocardial hypo perfusion scope of CHD patients with multiple coronary artery lesion is larger, and ischemia and anoxia are more severe. As a result, haemodynamical load of cardiac ventricle further increases, and abnormal load conditions further worsen. Besides, left ventricle dysfunction occurs and the functions present the spiral decline. Finally, progressive dilation of left ventricle is caused, and the disease worsens.^18^ The change of coronary plaque structure leads to coronary vascular cavity remolding, which is the compensatory response of blood vessels to blood flow resistance, artery wall damage and vascular endothelial cell proliferation. It can to some extent guarantee effective blood supply of diseased vessels and delay myocardial ischemia. But, positive remolding leads to Lumen eccentricity and plaque stress increase, and the plaque can easily fracture.^19^ The changes in some metabolism factors such as postprandial metabolism can also increase blood coagulation, thus leading to the increase of cardiovascular event risk, and unstable clinical manifestations of CHD patients.^20^,^21^ Linear correlation analysis showed that coronary artery lesion was positively correlated with LVEF and calcified plaque (P<0.05). Multiple linear regression analysis indicated that LVEF, calcified plaque and LDL-C were independent risk factors of multi-vessel coronary artery lesion of old CHD patients (P<0.05), which is consistent with previous research findings.^22^

In conclusion, the number of coronary artery lesions is significantly correlated with left ventricular remodeling, and can increase the proportion of calcified plaque and mixed plaque, thus leading to left ventricular remodeling abnormity.

### Authors’ contributions

**CW** & **XT:** Designed this study and significantly revised the manuscript.

**QL & WX:** Performed this study and drafted the manuscript.
